# Angiotensin-Converting Enzyme 2 Over-Expression in the Central Nervous System Reduces Angiotensin-II-Mediated Cardiac Hypertrophy

**DOI:** 10.1371/journal.pone.0048910

**Published:** 2012-11-14

**Authors:** Yumei Feng, Chetan Hans, Elizabeth McIlwain, Kurt J. Varner, Eric Lazartigues

**Affiliations:** 1 Department of Pharmacology and Experimental Therapeutics and Cardiovascular Center of Excellence, Louisiana State University Health Sciences Center, New Orleans, Louisiana, United States of America; 2 Department of Physiology, Tulane University, New Orleans, Louisiana, United States of America; 3 Center for Cardiovascular and Pulmonary Research, Nationwide Children's Hospital, Columbus, Ohio, United States of America; Max-Delbrück Center for Molecular Medicine (MDC), Germany

## Abstract

Angiotensin-converting enzyme type 2 (ACE2) has been shown to be an important member of the renin angiotensin system. Previously, we observed that central ACE2 reduces the development of hypertension following chronic angiotensin II (Ang-II) infusion in syn-hACE2 transgenic (SA) mice, in which the human ACE2 transgene is selectively targeted to neurons. To study the physiological consequences of central ACE2 over-expression on cardiac function and cardiac hypertrophy, SA and non-transgenic (NT) mice were infused with Ang-II (600 ng/kg/min, sc) for 14 days, and cardiac function was assessed by echocardiography. Blood pressure (BP), hemodynamic parameters, left ventricle (LV) mass/tibia length, relative ventricle wall thickness (2PW/LVD), cardiomyocyte diameters and collagen deposition were similar (*P*>0.05) between NT and SA mice during saline infusion. After a 2-week infusion, BP was elevated in NT but not in SA mice. Although ejection fraction and fractional shortening were not altered, Ang-II infusion increased 2PW/LVD compared to saline infusion in NT mice. Interestingly, the 2PW/LVD and LV mass/tibia ratios were significantly lower in SA compared to NT mice at the end of infusion. Moreover, Ang-II infusion significantly increased arterial collagen deposition and cardiomyocytes diameter in NT mice but not in transgenic animals (*P*<0.05). More importantly, ACE2 over expression significantly reduced the Ang-II-mediated increase in urine norepinephrine levels in SA compared to NT mice. The protective effect of ACE2 appears to involve reductions in Ang-II-mediated hypertension and sympathetic nerve activity.

## Introduction

The renin angiotensin system (RAS) plays an essential role in the physiological and pathological regulation of cardiovascular function and cardiac remodeling both at the periphery and in the central nervous system (CNS) [1]. A decade ago, a new member of the RAS, angiotensin-converting enzyme (ACE) type 2 was discovered from human heart failure ventricles and lymphoma cDNA libraries [2], [3]. Although ACE2 gene expression was first identified in various tissues such as kidney, heart, lung, and testis, it now appears that its expression is ubiquitous, including in the CNS and throughout brain regions involved in central regulation of cardiovascular function [4], [5]. In contrast to ACE, which generates Ang-II from Ang-I; ACE2, with an affinity ∼400 fold higher than for Ang-I, converts Ang-II into the vasodilatory peptide Ang-(1–7) [6]. In the CNS, Ang-II has been shown to promote sympathetic activity leading to vasoconstriction [1]. As a key enzyme for Ang-II degradation, ACE2 is thought to be a pivotal player in central regulation of cardiovascular function [7].

Several groups have shown that peripheral ACE2 has beneficial effects in the regulation of cardiac hypertrophy through degradation of Ang-II [8]–[10]. Additionally, systemic administration of a lentivirus coding for ACE2 protected rats from Ang-II-induced cardiac hypertrophy and fibrosis [11]. Finally, previous work from our group showed that ACE2 over-expression in the CNS blunted the development of Ang-II-induced hypertension and protected mice from Ang-II-induced baroreflex and autonomic dysfunctions [12]. However, the role of central ACE2 in the regulation of cardiac function and hypertrophy has not been addressed. In this study, using a transgenic mouse model over-expressing human ACE2 in the CNS, we investigated the role of central ACE2 in Ang-II-induced cardiac hypertrophy. Our data suggest that chronic ACE2 over-expression in the brain decreased Ang-II-mediated cardiac hypertrophy and collagen deposition, reduced urinary norepinephrine levels and partially protected SA transgenic mice from sympathetic -mediated cardiac hypertrophy and fibrosis.

## Materials and Methods

An expanded methods section is available in Materials and Methods S1.

### Animals

SA transgenic mice with neuron-targeted ACE2 over-expression, were generated as described previously [12]. Male SA transgenic and NT mice, 8–10 weeks old, were implanted subcutaneously with osmotic minipumps (Alzet) containing either saline or Ang-II (600 ng/kg/min) and infused for 14 days. All mice were fed standard mouse chow and water *ad libitum*.

### Ethics Statement

All procedures were approved by the Institutional Animal Care and Use Committee at the Louisiana State University Health Sciences Center and were in accordance with the National Institutes of Health Guidelines for the care and use of experimental animals.

### Telemetric blood pressure recording

The SA and NT mice were instrumented with radiotelemetric probes. Following recovery from surgery, baseline BP was recorded for 4 days. Mice were then infused subcutaneously with saline or Ang-II (600 ng/kg/min) for 14 days. BP was continuously recorded utill the end of the protocol as described [12].

### Echocardiography

Echocardiograms were performed at the end of the saline and Ang-II infusion protocol. Mice were under constant isoflurane (2% with 3 lpm O_2_) anesthesia. ECG electrodes were placed in a standard limb configuration to monitor heart rate. Ultrasound images were obtained with a Visualsonics VEVO 770 using a 30 MHz linear transducer. M-mode echocardiographic measurements of the interventricular septum (IS), LV posterior wall (LVPW) and LV internal diameter (LVID) were recorded in the parasternal short axis view at the level of the papillary muscles. LV systolic function was determined by fractional shortening (FS = LVD_diastole_−LVD_systole_/LVD_diastole_) and ejection fraction (EF). All measurements were performed on 3 different cardiac cycles and the values averaged.

### Left ventricle mass to tibia ratio measurement and histology

After a 2-week infusion of saline or Ang-II, mice were deeply anesthetized with Nembutal (50 mg/kg). Two minutes later, following verification of the lack of pedal reflex, mice were divided into 2 groups (n = 8/group). In the first group, left ventricles were dissected and the wet weight measured. Left tibia length was measured by using an electronic micrometer. Left ventricle mass/tibia ratios were calculated. In the second group, mice were perfused transcardially with PBS (0.1 M, pH 7.4) for 2 min followed by 10% formaldehyde in PBS (0.1 M, pH 7.4) for 10 min. Hearts and abdominal aorta tissue were collected and fixed in 10% formaldehyde/PBS overnight, dehydrated, embedded in paraffin and then sectioned. Hearts were cut in a cross section just below the level of the papillary muscle. The top half of the heart was formalin fixed and embedded in paraffin. Serial sections (5 µm) were prepared at 200 µm intervals. Sections were stained with hematoxylin and eosin for overall morphological examination, while Masson's Trichrome was used for collagen measurement. Images were quantified using Image-pro Plus software (Media Cybernetics, Inc. MD).

### Urine norepinephrine levels measurement

Urine was collected in tubes containing 6 N HCl at the end of the Ang-II and saline infusion protocol. Norepinephrine levels were measured using a CatCombi ELISA kit (IBL International, Hamburg, Germany) according to the manufacturer's instructions. The optical density was measured with a photometer at 405 nm (reference wavelength: 620–650 nm). Each sample was measured in triplicate.

### Statistical Analysis

Data are expressed as mean ±SEM. Data were analyzed by Student's *t* test or two way ANOVA (Bonferroni post hoc tests to compare replicate means) when appropriate. Statistical comparisons were performed using Prism5 (GraphPad Software, San Diego, CA). Differences were considered statistically significant at *P*<0.05.

## Results

### Brain-targeted ACE2 expression reduces Ang-II-induced cardiac hypertrophy

To determine the effects of Ang-II infusion on cardiac function, hemodynamic and cardiac parameters were measured by echocardiography as summarized in Table 1. hemodynamic parameters were not significantly different between NT and SA mice following saline infusion. Ang-II infusion significantly decreased the left ventricle internal diameter (LVID) and left ventricle volume (LV Vol) in NT and SA compared to saline infusion (P<0.05). However, there was no difference in LVID or LV Vol between NT and SA mice following Ang-II infusion. NT mice exhibited a significant increase in left ventricle posterior wall thickness (LVPW) following 2 weeks of Ang-II infusion compared to saline-treated NT mice (*P*<0.05). Ang-II infusion did not change the LVPW in SA mice and the LVPW was significantly smaller in SA compared to NT mice at the end of the 2 weeks Ang-II infusion (*P*<0.05).

**Table 1:** Cardiac hemodynamic parameters for NT and SA mice.

Measurement (units)	NT Saline	SA Saline	NT Ang II	SA Ang II
BP (mmHg)	95±1	98±2	136±3#	110±4*
HR (bpm)	451±11	489±10	562±21#	527±12
IVS; d mm	0.68±0.05	0.77±0.03	0.77±0.07	0.65±0.02
IVS: s mm	1.06±0.03	1.21±0.07	1.11±0.05	1.07±0.06
LVID; d mm	4.66±0.11	4.79±0.10	4.11±0.11#	4.13±0.14#
LVID; s mm	3.62±0.12	3.74±0.13	3.12±0.10#	3.28±0.12#
LVPW; d mm	0.73±0.02	0.67±0.03	0.86±0.02#	0.66±0.03*
LVPW;s mm	1.02±0.03	0.92±0.04	1.10±0.06#	0.91±0.05*
LV Vol; d ul	101.0±5.97	107.2±5.97	75.21±5.96#	76.41±5.78#
LV Vol; s ul	55.43±4.81	60.43±4.96	38.94±2.83#	44.30±3.63#
%EF	45.05±3.03	43.67±3.62	47.45±3.53	41.74±3.88
% FS	22.53±1.84	21.85±2.19	23.75±2.16	20.47±2.15

The data is expressed as mean ± SEM (n = 6). All measurements were performed on 3 different cardiac cycles and the values averaged. BP: blood pressure; HR: heart rate; IVS: interventricular septum; LVID: left ventricle internal diameter; LVPW: left ventricle posterior wall thickness; LV Vol: left ventricle end volume; %EF: ejection fraction; %FS: *P* value (<0.05).

* compared to NT in the same treatment and

# compared to saline in the same genotype.

**Figure 1:**
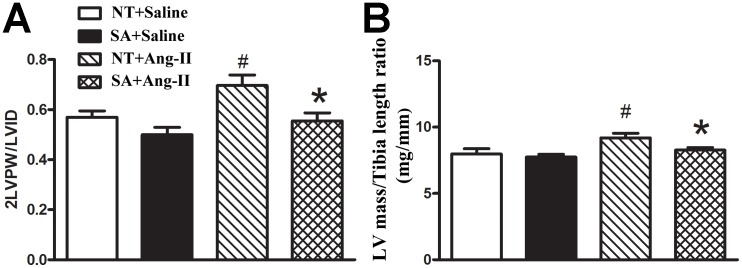
ACE2 over expression decreases Ang II-induced left ventricle cardiac hypotrophy. 2 weeks after Ang II or saline infusion, echocardiography were performed on 3 different cardiac cycles, the values averaged and used to calculate 2PW/LVD; then, mice left ventricle were collected and tibia length were measured for calculation of LV mass/Tibia ratio. ACE2 over expression significantly reduced the increase of 2PW/LVD (**A**) and LV mass/Tibia ratio (**B**) in SA compare to NT mice following Ang II infusion (P<0.05). N = 8/group. Statistical significance: *P<0.05 vs. NT with the same treatment; ^#^ *P*<0.05 vs. saline with the same genotype.

To examine the type of hypertrophy the hearts had undergone, we considered the relative wall thickness (RWT) by calculating 2× LVPW/LVID. As shown in Figure 1A, RWT was similar between NT and SA mice (0.56±0.03 vs. 0.50±0.03, P>0.05) following saline infusion. Ang-II infusion significantly increased the 2LVPW/LVID (0.70±0.04, P<0.05) compared to saline-infused animals in NT mice; while it remained normal in SA+Ang-II mice (0.56±0.03, P>0.05). As another parameter defining cardiac hypertrophy, the left ventricle wet weight to tibia length ratio (LV mass/tibia length ratio) was calculated directly after mice were sacrificed (Figure 1B). Similarly, there is no difference between NT and SA mice at following saline infusion (7.98±0.41 vs. 7.75±0.21, P>0.05). Ang-II infusion significantly increased the LV mass/tibia length ratio in NT+Ang-II mice compared to NT+Saline (9.20±0.35, P<0.05) and SA+Ang-II mice (8.27±0.19, P<0.05). Consequently, the LV mass/Tibia length ratio was significantly lower in SA compared to NT after Ang-II infusion (P<0.05).

**Figure 2:**
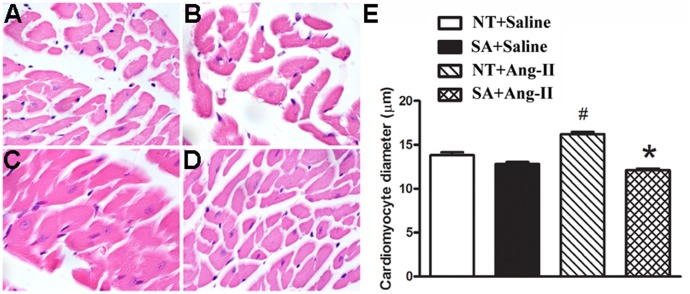
Brain-targeted ACE2 over expression prevents the increase in cardiomyocyte diameters following Ang II infusion. Mice were anesthetized and hearts were processed for H&E staining at the end of 2 weeks Ang II or saline infusion. The average cardiomyocyte diameters were quantized by using Image-Pro Plus software. The cardiomyocyte diameters were not different between NT (**A**) and SA (**B**) mice following 2 weeks of saline infusion (P>0.05). Ang II infusion significantly increased the cardiomyocyte diameters in NT (**C**) compared to saline infusion (P<0.05). However, it was smaller in SA (**D**) compared to NT mice at the end of 2 weeks Ang II infusion (P<0.05). N = 5/group. Statistical significance: *P<0.05 vs. NT with the same treatment; ^#^ *P*<0.05 vs. saline with the same genotype.

The effect of Ang-II infusion on cardiac hypertrophy was examined by measuring myocyte cross-sectional diameter on H&E stained cardiac sections. Histology of the heart was performed by an expert pathologist who was blinded to the study, showed (Figure 2) that Ang-II infusion significantly increased cardiomyocytes diameter compared to saline infusion in NT mice (16.20±0.26 µm vs. 13.80±0.34 µm, P<0.05). More importantly, the cardiomyocytes diameter was smaller in SA (Figure 2D) hearts compared to NT mice (Figure 2C) following Ang-II infusion (12.10±0.19 µm, P<0.05), as shown in the quantified data (Figure 2E). These data suggest that ACE2 over-expression in the brain decreased the Ang-II-induced cardiac hypertrophy in SA mice.

### ACE2 over-expression in the brain decreases collagen deposition in coronary arteries and the abdominal aorta

**Figure 3:**
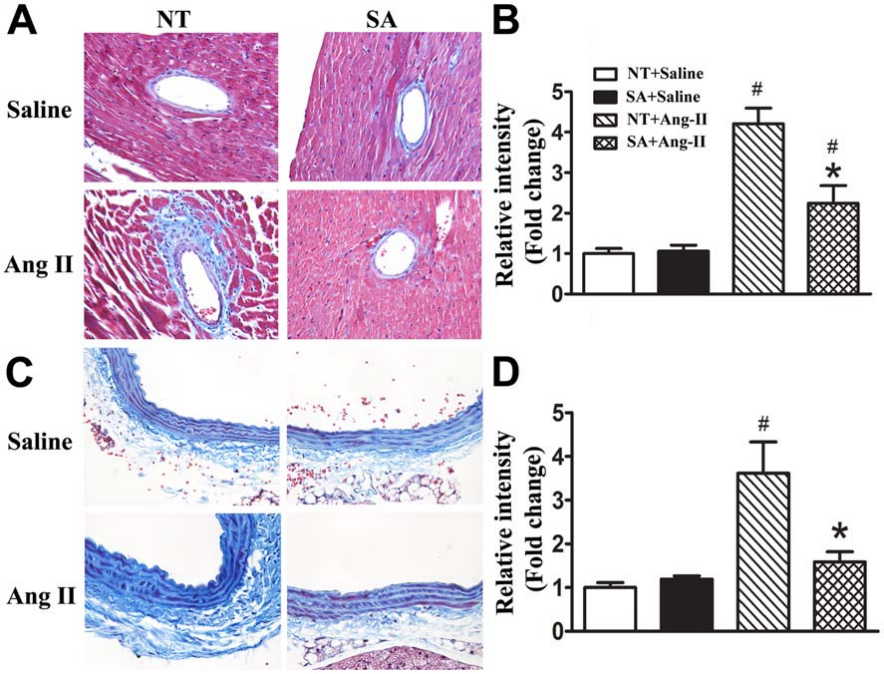
ACE2 over expression in the brain reduces Ang II-induced collagen deposition in aortas and coronary arteries. Mice were anesthetized and hearts were processed for Masson's Trichrome staining at the end of 2 weeks Ang II or saline infusion. The average trichrome staining densities were quantified by using Image-Pro Plus software. Brain-targeted ACE2 over expression significantly reduces collagen deposition in coronary arteries (**A, B**) and aortas (**C, D**) following 2 weeks of Ang II infusion. N = 5/group. Statistical significance: *P<0.05 vs. NT with the same treatment; ^#^ *P*<0.05 vs. saline with the same genotype.

To investigate the role of central ACE2 in the regulation of end organ damage following Ang-II infusion, we examined collagen deposition in coronary arteries and abdominal aortas by Masson's Trichrome staining (Figure 3). Collagen deposition was similar between NT (1.0±0.11; 1.0±0.12) and SA (1.19±0.07; 1.05±0.15) mice in coronary arteries and aortas, respectively following saline infusion. Ang-II infusion significantly increased collagen deposition in both coronary arteries (4.21±0.38) and aortas (3.61±0.71) compared to NT+Saline mice (P<0.05). However, SA+Ang-II had significantly lower collagen deposition in both coronary arteries (2.24±0.44) and aortas (1.59±0.23) compared to NT mice following Ang-II infusion. All together, these data suggest that brain-targeted ACE2 over-expression reduces the collagen deposition in coronary arteries and aorta, indicating a protective effect for central ACE2 during cardiac fibrosis events.

### ACE2 overexpression in brain decreases urine norepinephrine levels in response to Ang-II

**Figure 4:**
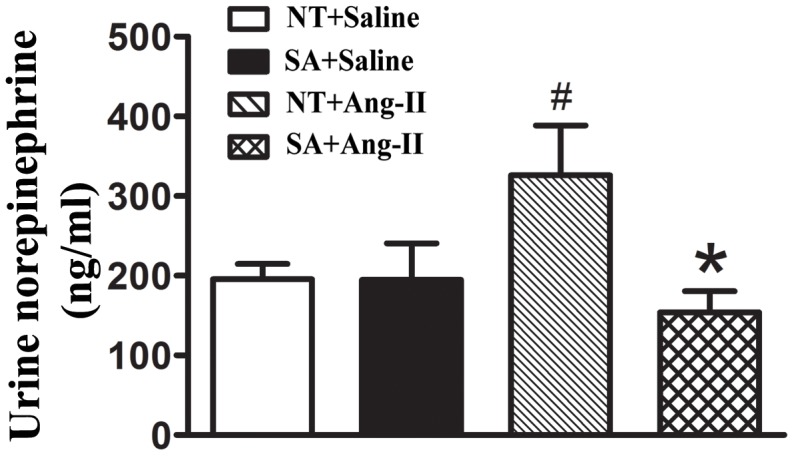
Urine NE levels. Mice were infused with Ang II or saline for 2 weeks. Urine were collected in 6 N HCl and processed for NE measurements using ELISA methods. Two weeks of Ang II infusion significantly increase urine NE levels compared to saline infusion in NT mice. However, the NE levels is significantly lower in SA compared NT at the end of 2 weeks infusion. N = 4/group. Statistical significance: *P<0.05 vs. NT with the same treatment; ^#^ *P*<0.05 vs. saline with the same genotype.

Release of norepinephrine (NE) from peripheral nerves into the circulation modulates the level of sympathetic tone and contributes to cardiac hypertrophy. To clarify the involvement of this mechanism in the reduction of cardiac hypertrophy in SA mice, urine NE levels were assessed (Figure 4). The NE levels were similar between NT (195±19 ng/ml) and SA (195±46 ng/ml) mice following saline infusion (P>0.05). As expected, Ang-II infusion significantly increased NE levels compared to saline-infused NT mice. Interestingly, NE levels were not increased in SA+Ang-II mice compared to SA+saline. Moreover, it was 50% lower in SA (154±27 ng/ml) compared to NT mice (326±62 ng/ml) at the end the 2-week Ang-II infusion (P<0.05). The data suggest that over-expression of ACE2 in the CNS reduces sympathetic activity, as illustrated by a decrease in urinary NE levels.

## Discussion

As an important regulator of blood pressure and cardiovascular function, the RAS can cause cardiac hypertrophy and fibrosis by increasing ventricular wall stress or direct trophic action of Ang-II, one of the major actors in this system [13], [14]. In addition to the systemic RAS, a local RAS has been identified in various tissues, including the brain [15]. The brain RAS contains the same elements as the other tissue RAS and has long been considered pivotal for cardiovascular regulation as well as in the pathogenesis of hypertension and cardiac diseases [15], [16]. Most of the brain nuclei are inside the blood brain barrier and are therefore protected from systemic neuromediators. However, some nuclei, called circumventricular organs (CVO), are lacking a blood brain barrier and, as a result, constitute “open windows” to the brain for small peptides, like Ang-II [17]. Therefore, in addition to locally-generated Ang-II in the CNS, blood-borne Ang-II can reach the brain via the CVO and interact with angiotensin receptors located in these areas to exert central effects in addition to its peripheral effects [4], [18]. The consequences are alteration of sympathetic and parasympathetic tone, leading to the modulation of NE release and cardiac function. As an important member of the RAS, brain ACE2 has been shown to counterbalance the effects of the ACE/Ang-II/AT1 receptor axis and to play a pivotal role in the maintenance of BP [19]–[22]. However, the question of whether ACE2 in the brain can similarly regulate cardiac function and hypertrophy has not yet been investigated. During the past 2 decades transgenic models have proven to be valuable tools for dissecting the function of the brain RAS [1]. Using a transgenic mouse model, over-expressing hACE2 in the CNS [12], we investigated the role of central ACE2 in Ang-II-induced cardiac hypertrophy. Our data suggest that chronic ACE2 over-expression in the brain decreased the Ang-II-induced cardiac hypertrophy of the LV and collagen deposition in the coronary vessels and aorta, and reduced NE release.

Recently, several studies have focused on the beneficial effects of peripheral ACE2 in the regulation of cardiac hypertrophy [8]. Zhong *et al.* were the first to show an association between reduction of BP and increased ACE2 mRNA and protein in heart and kidneys of SHR. More importantly, these decreases were associated with left ventricular hypertrophy in SHR [10]. In addition, Kaiqiang et al. showed that olmesartan improved LV function and hypertrophy through the increase of ACE2 mRNA in pressure-overloaded rat hearts [23]. Further evidence of the protective role of ACE2 against cardiac hypertrophy and fibrosis was demonstrated following lentivirus-mediated over-expression of ACE2 in the heart [9], [11]. Moreover, 2 independent ACE2 polymorphism studies indicated that genetic variants in the ACE2 gene may be associated with LV mass and LV hypertrophy in hemizygous men [24], and minor alleles of ACE2 gene might be the genetic modifier for the magnitude of LV hypertrophy in male patients with hypertrophic cardiomyopathy [25]. These studies support the involvement of ACE2 in cardiac hypertrophy at the gene level.

These SA mice used in this study have normal resting hemodynamic parameters. Importantly, brain-targeted ACE2 over-expression blunts neurogenic hypertension induced by the ‘slow pressor dose’ Ang-II model, partially by preventing the decrease in both spontaneous baroreflex sensitivity and parasympathetic tone [20]. In this model, infusion of a low dose of Ang-II is most effective at reaching the brain, via the CVO, and acting on nuclei controlling BP rather than directly affecting peripheral vasculature [26], therefore leading to increased sympathetic outflow. Although 2 weeks of ‘slow pressor dose’ Ang-II infusion did not impair cardiac function in either SA or NT mice, our data show that ACE2 over-expression in the brain reduced LV mass and the relative wall thickness at the end of Ang-II infusion. As we reported previously [20], over-expression of ACE2 in the CNS converts brain Ang-II into Ang-(1–7), leading to a reduction of Ang-II in the brain of these mice. Moreover, the Ang II/Ang-(1–7) peptides ratio was significantly decreased in SA mice. All the evidence suggested a lower activation of the brain ACE/Ang-II/AT1 receptor axis, a decrease in sympathetic outflow following Ang-II infusion and supported a reduction of neurogenic hypertension in SA mice.

Sympathetic overdrive has been shown to result in cardiac hypertrophy and fibrosis [27]. Ang-II has an effect both on the CNS which would enhance sympathetic outflow, and on the peripheral sympathetic nerves [28]. Moreover, it is well known that Ang-II has a direct effect on activating cardiac myocyte and myofibroblast signaling pathways, resulting in cardiac and vascular hypertrophy and fibrosis [29]–[31]. In this study, we observed an increase in cardiac myocyte diameters and vascular collagen deposition in NT mice following Ang-II infusion, consistent with previous reports [29], [32]. More interestingly, we found that brain-targeted ACE2 over-expression reduced Ang-II-induced cardiac hypertrophy and fibrosis. Since hACE2 expression is limited to the CNS in SA mice, the protective effects might be partly due to the reduction in BP and the modification of sympathetic and parasympathetic tones in this model [20]. This is consistent with previous data showing that sympathetic activity correlated with myocardial hypertrophy in patients with hypertrophic cardiomyopathy [33], [34].

Cardiac hypertrophy starts as an adaptive response of the heart against hemodynamic overload or neurohumoral factors [35]. It is well recognized that Ang-II and NE activation of independent signaling pathways are major mechanisms leading to cardiac hypertrophy [36]. Furthermore, Ang-II induces NE release from cardiac nerve fibers through interaction with AT1 receptors located in the central and peripheral nervous systems [37], [38]. Interestingly, we observed that ACE2 over-expression in the brain reduced urinary NE levels, an indirect indication of reduced sympathetic outflow. This reduction of sympathetic drive may at least partially contribute to the protection from cardiac hypertrophy and fibrosis in SA mice following Ang-II infusion. In fact, several studies have shown that cardiac hypertrophy is associated with sympathetic change and an increase in NE levels, which further support our results [39], [40].

In summary, brain-targeted ACE2 over-expression plays a pivotal role in the regulation of cardiac function. ACE2 over-expression in the brain reduces both the Ang-II-induced cardiac collagen deposition and cardiomyocyte hypertrophy. The mechanism by which this occurs appears to involve at least a decrease in NE release and reduction of BP. Therefore, we conclude that ACE2 over-expression reduces Ang-II-induced cardiac hypertrophy partially through a decrease in sympathetic drive in SA transgenic mice.

## Supporting Information

Materials and Methods S1 (DOCX)
