# Supplementary material for: Angiotensin-Converting Enzyme 2 Over-Expression in the Central Nervous System Reduces Angiotensin-II-Mediated Cardiac Hypertrophy
Source: PLoS One. 2012 Nov 14;7(11):e48910. doi: 10.1371/journal.pone.0048910 (PMC3498357; doi:10.1371/journal.pone.0048910)
Supplement: Materials and Methods S1 — (DOCX) [file pone.0048910.s001.docx]

**Online Data Supplements**

**Material and Methods**

**Animals**

The SA transgenic mice were generated as described.{Feng, 2009 #2496} Male SA transgenic and NT mice (n=8), 8-10 weeks old, were anesthetized and infused subcutaneously for 14 days using osmotic minipumps (Alzet) containing either saline or Ang-II (600ng/kg.min). All mice were fed standard mouse chow and water *ad libitum*. All procedures were approved by the Institutional Animal Care and Use Committee at the Louisiana State University Health Science Center.

**Echocardiography**

Male SA and NT mice (n=8), 8-10 weeks old, were anesthetized and infused subcutaneously for 14 days using osmotic minipumps (Alzet) containing either saline or Ang-II (600 ng/kg.min). Echocardiograms were performed at the end of infusion during the protocol.  Mice were anesthetized with isoflurane (2% with 3lpm O_2_).  ECG electrodes were placed in a standard limb configuration to monitor heart rate. Ultrasound images were obtained with a Visualsonics VEVO 770 using a 30 MHz linear transducer. M-mode echocardiographic measurements of the interventricular septum (IS), posterior wall (PW) and LV diameter (LVD) were recorded in the parasternal short axis view at the level of the papillary muscles. LV systolic function was determined by fractional shortening (LVD_diastole_-LVD_systole_/LVD_diastole_). All measurements were performed on 3 different cardiac cycles and the values averaged.

**Left ventricle mass to tibia ratio measurement**

Male SA and NT mice (n=8), 8-10 weeks old, were anesthetized and infused subcutaneously for 14 days using osmotic minipumps (Alzet) containing either saline or Ang-II (600 ng/kg.min). At the end of two weeks infusion, Mice were deeply anesthetized with Nembutal (10ug/kg) and sacrificed. Left ventricles were dissected and the wet weight measured. Left tibia length was measured by using an electronic micrometer. Left ventricle mass/Tibia ratios were calculated.

**Histogy: H&E staining and Masson’s Trichrome staining**

Male SA and NT mice (n=8), 8-10 weeks old, were anesthetized and infused subcutaneously for 14 days using osmotic minipumps (Alzet) containing either saline or Ang-II (600 ng/kg.min). At the end of two weeks infusion, Mice were deeply anesthetized with Nembutal (10ug/kg) and perfused transcardially with PBS (0.1M, pH 7.4) for 2 min followed by 10% formaldehyde in PBS (0.1M, pH 7.4) for 10 min. Hearts and abdominal aorta tissue was collected and fixed in 10% formaldehyde/PBS overnight, dehydrated, and embedded in paraffin then sectioned. Hearts were cut in a cross section just below the level of the papillary muscle. The top half of the heart was formalin fixed and embedded in paraffin. Serial sections (5 µm) were prepared at 200-µm intervals. The sections were stained with hematoxylin and eosin for overall morphological examination, while Masson’s Trichrome was used for collagen measurement. Images were quantized by using Image-pro Plus software (Media Cybernetics, Inc. MD).

**Urine norepinephrine levels measurement**

Male SA and NT mice (n=8), 8-10 weeks old, were anesthetized and infused subcutaneously for 14 days using osmotic minipumps (Alzet) containing either saline or Ang-II (600 ng/kg.min). Urine was collected in the presence of 6N HCl at the end of two weeks of Ang II infusion. The norepinephrine levels were measured by using a CatCombi ELISA kit per the instructional manual (IBL International, Hamburg, Germany). As described, each Standard, Control and urine samples (5 fold diluted in 0.1 N HCl) were added into the respective wells of the extraction plate in extraction buffer and extracted 30 min at room temperature on an orbital shaker (600rpm). Plate was washed with bidistilled water at room temperature for 5 min on an orbital shaker (600rpm). Samples were then acylated in acylation reagent containing dimethylformamide and ethanol for 20 min and washed with bidistilled water. Following acylation, samples were released in the release buffer containing 0.1 N HCl for 30 min at room temperature and used for norepinephrine ELISA. For ELISA, samples were incubated with coenzyme solution and norepinephrine antiserum for 120 min at room temperature and washed 4 times in wash buffer. Samples then were incubated with secondary antibody conjugated to alkaline phosphatase for 60 min and washed 4 times in wash buffer. Reactions were developed by adding alkaline phosphatase substrate p-nitrophenyl phosphate. The optical density was measured with a photometer at 405 nm (reference wavelength: 620-650). Each sample was measured in triplicate.

**Statistical Analysis**

Data are expressed as mean ±SEM. Data were analyzed by Student’s *t* test or two-way ANOVA (Bonferroni post hoc tests to compare replicate means) when appropriate. Statistical comparisons were performed using Prism5 (GraphPad Software, San Diego, CA). Differences were considered statistically significant at *P<*0.05.
